# FcRN receptor antagonists in the management of myasthenia gravis

**DOI:** 10.3389/fneur.2023.1229112

**Published:** 2023-08-04

**Authors:** Vinaya Bhandari, Vera Bril

**Affiliations:** Ellen and Martin Prosserman Centre for Neuromuscular Diseases, Toronto General Hospital, University Health Network, University of Toronto, Toronto, ON, Canada

**Keywords:** neonatal Fc receptor (FcRn), myasthenia gravis (MG), immunoglobulins (IgG), clinical trials, Fc receptor inhibitors

## Abstract

Myasthenia gravis (MG) is an autoimmune disorder characterized by autoantibodies specifically directed against proteins located within the postsynaptic membrane of the neuromuscular junction. These pathogenic autoantibodies can be reduced by therapies such as plasma exchange, IVIG infusions and other immunosuppressive agents. However, there are significant side effects associated with most of these therapies. Since there is a better understanding of the molecular structure and the biological properties of the neonatal Fc receptors (FcRn), it possesses an attractive profile in treating myasthenia gravis. FcRn receptors prevent the catabolism of IgG by impeding their lysosomal degradation and facilitating their extracellular release at physiological pH, consequently extending the IgG half-life. Thus, the catabolism of IgG can be enhanced by blocking the FcRn, leading to outcomes similar to those achieved through plasma exchange with no significant safety concerns. The available studies suggest that FcRn holds promise as a versatile therapeutic intervention, capable of delivering beneficial outcomes in patients with distinct characteristics and varying degrees of MG severity. Efgartigimod is already approved for the treatment of generalized MG, rozanolixizumab is under review by health authorities, and phase 3 trials of nipocalimab and batoclimab are underway. Here, we will review the available data on FcRn therapeutic agents in the management of MG.

## Introduction

MG represents an autoimmune disorder characterized by autoantibodies specifically directed against proteins located within the postsynaptic membrane of the neuromuscular junction (1, 2). This results in the development of focal or generalized muscle weakness in the skeletal muscles. The clinical spectrum encompasses a variety of manifestations, ranging from isolated ocular involvement to profound weakness affecting the limbs, bulbar region, and respiratory muscles. The weakness is fatigable and fluctuating in nature, improving with rest. The prevalence of MG, a relatively uncommon disease, ranges from 5.3 to 35 cases per 100,000 individuals, while the incidence ranges from 0.3 to 2.8 new cases per 100,000 individuals (3–5). Over the past few decades, the prevalence of MG has shown a steady increase, attributed to factors such as improved diagnostic capabilities, advancements in therapeutic options, and increased life expectancy of MG patients.

## Pathogenesis of myasthenia gravis

In normal neuromuscular transmission, the presynaptic membrane releases acetylcholine (ACh) which then binds to the acetylcholine receptor (AChR) situated on the postsynaptic membrane. This interaction produces an end plate potential (EPP), the magnitude of which is dictated by the quantity of ACh released at the presynaptic membrane and its interaction with the receptor. Under normal circumstances, the EPP rises above the depolarization threshold to produce an action potential and muscle contraction. In MG, impaired neuromuscular transmission and reduced safety factor (EPP) amplitude reduces muscle contraction (6, 7). About 80% of patients with MG in some series have anti-acetylcholine receptor antibodies (anti-ACHR ab) (8, 9). The prevalence of the IgG1 and IgG3 subclasses of antibodies is most observed in patients diagnosed with myasthenia gravis (10). These autoantibodies bind to AChR at the terminal expansions of the junctional folds that cause activation of the complement system forming membrane attack complexes (MAC), causing accelerated internalization, degradation of ACHR and destruction of the ACHR receptors. In addition, there is cross-linking of the autoantibodies, causing a conformational change in the ACHR receptor, which also interferes with neuromuscular transmission (11). Another mechanism affecting the neuromuscular junction in MG is the functional blockade of ACHR receptors by antibodies and the disruption of junctional folds. This causes the postsynaptic membrane to be distorted and simplified (12–15). These processes lead to neuromuscular failure, muscle weakness and characteristic worsening with an extended period of activity. There is a decremental response of the motor unit potential to repetitive nerve stimulation (RNS) as there is a reduction in safety factor. Up to 50% of patients with anti-ACHR negative generalized MG (ACHR- gMG) may have circulating antibodies against muscle-restricted receptor tyrosine kinase (MuSK), constituting about 5% of the total generalized MG cases. These are mainly IgG4 antibodies and do not affect the complement system. These antibodies mainly affect the clustering of the ACHR, thereby causing a functional blockage at the neuromuscular junction (16, 17). Roughly 2% of individuals diagnosed with double seronegative generalized myasthenia gravis (gMG) display low-density lipoprotein receptor-related protein 4 antibodies (anti-LRP antibodies) (18). The IgG1 subclass accounts for most anti-LRP (lipoprotein receptor-related protein) antibodies and causes damage to the postsynaptic function by complement-mediated damage and possibly interfering with agrin-induced MUSK activation (19, 20). A proportion of patients with MG have a presence of low-affinity antibodies, which cannot be detected by routine radioimmunoassays (RIAs) and can be detected by specific assays such as cell-based assays (21).

MG does not follow a classic Mendelian inheritance pattern, indicating that it is not a hereditary disease. However, there is an increased likelihood of family members of MG patients developing the disease compared to the general population (22). Studies have shown a concordance rate of 35% in monozygotic twins and 5% in dizygotic twins, suggesting a genetic contribution to MG pathogenesis. Nonetheless, environmental factors also play a crucial role (23). Various HLA types, such as DR2, DR3, B8, and DR1, are associated with an increased predisposition to MG.

The breakdown of mechanisms responsible for maintaining immune tolerance to self-antigens is the underlying cause of autoimmunity. During development, most auto-reactive T cells are eradicated in the thymus, establishing central tolerance. Regulatory mechanisms involved in maintaining immune tolerance encompass the generation of regulatory T cells (Treg cells), which exert control over self-reactive T cells that escape thymic elimination. Essential roles in the clonal deletion and Treg cell selection are performed by transcription factors, notably the autoimmune regulator gene (AIRE) (24). The thymus is pivotal in MG pathogenesis, as 85% of patients exhibit thymic gland abnormalities, with 70% showing thymic hyperplasia and 10% having a thymoma. The regulation of autoreactive T cells is impaired due to thymic hyperplasia or thymoma, possibly due to altered expression of AIRE and inefficient Treg generation in patients with MG (25). There is a breakdown of immune tolerance to self-antigens that leads to autoimmunity.

The pathophysiology of MG is complex and multifactorial, attributed to the interplay between genetic and environmental factors leading to complex immune-mediated dysfunction at the neuromuscular junction.

There is upregulation of CD4+ T cells due to the breakdown of immune tolerance, leading to the release of various proinflammatory cytokines (IL-2, IL-4 and IL-6), leading to B cell stimulation, eventually leading to antibody production (5). As a consequence of these mechanisms, mature T and B lymphocytes in the thymus gland become activated. The activation of T cells results in the secretion of proinflammatory cytokines like IFN-gamma and IL-17, leading to an imbalance between regulatory T cells (Treg), which are deficient, and hyperactivated Th17 cells, further amplifying antibody production in MG (26–28).

## Conventional treatment in myasthenia gravis

The treatment of MG ranges includes various non-immunosuppressive, immunosuppressive therapeutic agents and newer biological agents which are target specific (29). The acetylcholinesterase agents such as pyridostigmine provide symptomatic treatment of myasthenia gravis; however, seldom as sole therapy. In older literature, about 30–50% of MG patients were reportedly on pyridostigmine alone (30). Corticosteroids likely act by inhibiting T lymphocytes and monocyte–macrophage activation and are used in many MG patients (31). Azathioprine, mycophenolate mofetil, tacrolimus, methotrexate, and cyclophosphamide are some of the immunosuppressive agents used in the treatment of MG include, and these act by varied mechanisms that suppress the immune system. The main role of immunosuppressive agents is to act as steroid-sparing agents and to prevent the long-term side effects caused by corticosteroids, which include diabetes mellitus, weight gain, cataracts, hypertension, osteoporosis, and gastric ulcer (32). However, the immune suppressive agents have their own set of side effects and increase the propensity to cause infection and cancers such as squamous cell carcinomas.

IVIG and PLEX have proven to be efficacious in acute exacerbations of MG and MG crises (33, 34). IVIG is more widely available and has fewer side effects than PLEX. Several studies have shown no difference in the overall efficacy of IVIG over PLEX (35, 36). In cases of chronic refractory disease or when patients do not respond to standard immunosuppressant therapy, intravenous immunoglobulin (IVIG) and subcutaneous immunoglobulin (SCIG) Infusions have shown beneficial effects (37). However, the management of IVIG has its own challenges. Various side-effects associated with IVIG administration include aseptic meningitis, headaches, increased propensity to thrombosis, and renal impairment (38).

PLEX is a therapeutic approach employed in the treatment of myasthenia gravis since 1976 (39). This includes the elimination of pathogenic and normal immunoglobulins and high molecular weight components like albumin and proinflammatory factors that contribute to the autoimmune process. The effects of PLEX last for 2–4 weeks (40). PLEX requires specialized equipment, central venous access, and nurse supervision. As coagulation factors are removed during PLEX treatment, it is performed on alternate days to allow natural recovery. However, tissue IgG is redistributed between the PLEX sessions, and the serum IgG rises again. Thus, there is a need for agents that mimic the role of PLEX or IVIG but have a sustained effect with fewer side effects.

## Fc gamma receptors (FcγR) and neonatal Fc receptors (FcRn) as therapeutic agents

Antibodies constitute the most important part of adaptive immunity. Immunoglobulin G (IgG) is the predominant class of antibodies, accounting for approximately 75–80% of the total immunoglobulin pool (41). These antibodies can be found in both circulation and extracellular fluids. Immunoglobulins exhibit a structural arrangement composed of two heavy chains and two light chains, giving rise to a molecular configuration encompassing two fragment antigen binding domains (Fab) and a glycosylated crystallizable fragment (Fc). The hinge region connects the two Fab fragments and the Fc fragment, enabling conformational flexibility to the Fab fragment. Because the Fab fragments are identical, they can bind to specific target antigens (42–44). The effector function of the Fc region is facilitated through its interaction with various receptor molecules such as Fc gamma receptors (FcγR) and the first subcomponent of the C1 complex (C1q), facilitating crucial functions such as induction of mediator secretion, antibody-dependent cellular cytotoxicity (ADCC), antibody-dependent cellular phagocytosis (ADCP), endocytosis of opsonized particles, complement-dependent cytotoxicity (CDC) (45, 46).

FcγR is a diverse family of proteins encompassing classical membrane-bound surface receptors, atypical intracellular receptors, and cytoplasmic glycoproteins. Cells of hemopoietic origin widely express receptors and can be either activating receptors (FcγRI, FcγRIIA, FcγRIIC, FcγRIIIA, and FcγRIIIB) or inhibitory receptors (FcγRIIB) and differ in their affinity to various IgG subclasses (47) FcRn belongs to the FcγRs is structurally unique and differs from the classic members of the receptor family in various aspects. It was initially discovered that the transfer of maternal antibodies to neonates was possible due to proteins called neonatal Fc receptors (FcRn) (48). FcRn is a beta-2 macroglobulin-associated protein exhibiting structural similarity to the major histocompatibility class I (MHC-I) family. FcRn is monomorphic, quasi-ubiquitously expressed, and expressed in various body tissues, including epithelia, endothelia, hemopoietic cells, intestinal cells, kidney, liver, and liver placenta (49–53).

Amongst all the serum proteins, albumin and IgG have the longest half-life period of approximately 3 weeks compared to other serum proteins, which have a half-life period of approximately 5–7 days (54). FcRn uniquely binds both IgG and albumin, despite the structural and functional dissimilarity between these two molecules. Intracellularly, FcRn binds to IgG and albumin at non-overlapping sites within endosomes at pH 5–6.5. It then prevents the catabolism of IgG and albumin by preventing their lysosomal degradation and releasing them outside the cell at physiologic pH, thus prolonging the half-life of the albumin and IgG (55–58).

**Figure 1:**
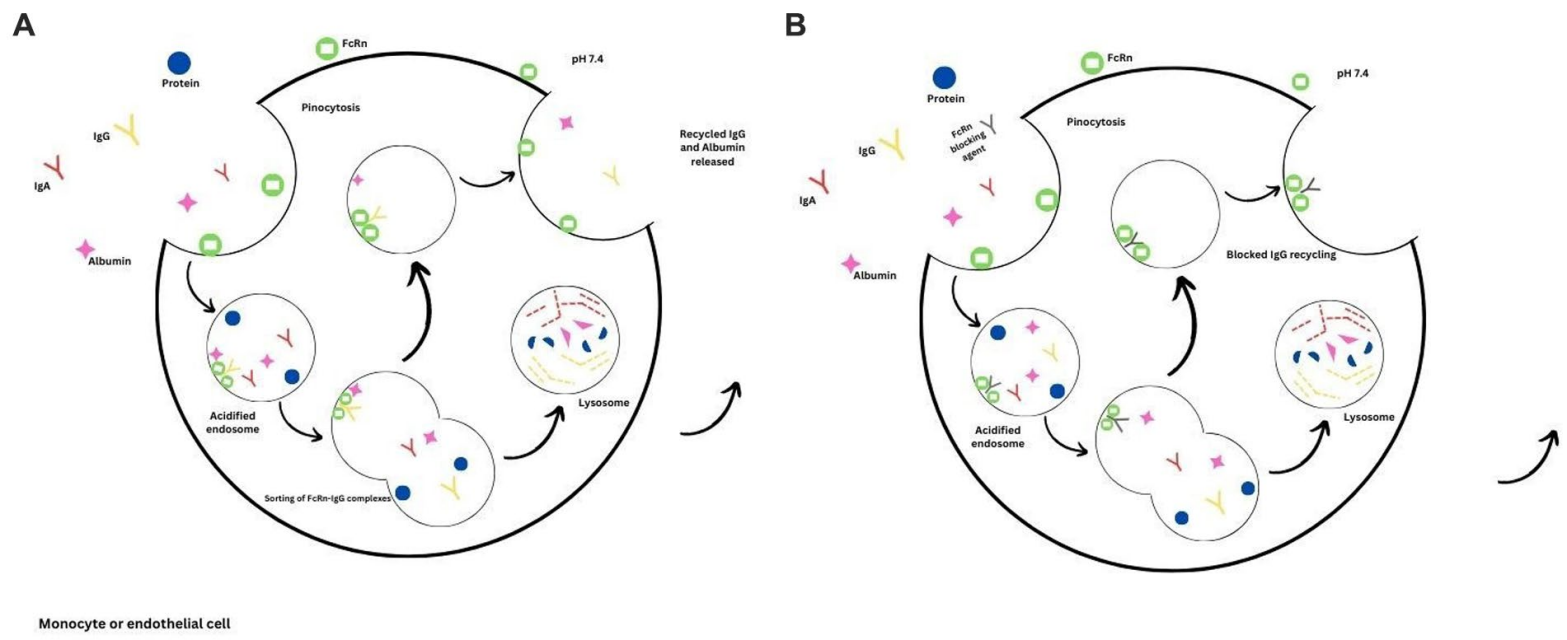
FcRn mediated recycling. **(A)** The binding of IgG and albumin to the FcRn receptors prolongs their half-lives due to the prevention of lysosomal degradation. **(B)** Binding of the FcRn therapeutic agent, thus preventing the recycling of IgG due to increased lysosomal degradation.

The presence of pathogenic autoantibodies characterizes autoimmune disorders such as MG. Current therapies such as plasma exchange, IVIG infusions and other immunosuppressive agents aim to reduce the pathogenic antibodies. However, most of these therapies exhibit a broader mechanism of action and have significant side effects. Since there is a better understanding of the molecular structure and its biological properties, the FcRn possesses an attractive profile in treating myasthenia gravis and other autoimmune disorders whereby the autoantibodies can be reduced by blocking the FcRn. By blocking the FcRn, the catabolism of IgG will be enhanced, and similar results will be achieved with plasma exchange (Figure 1). This mechanism will be useful in various autoimmune disorders; degradation of IgG molecules can be achieved by blocking the FcRn receptors is a rational therapeutic approach (59, 60).

## FcRn therapeutic agents in the management of MG

Table 1 gives an overview of the various FcRn blocking agents developed in the management of MG. The remainder of this article focuses on the evidence for these agents in the treatment of MG.

**Table 1:** Overview of the FcRn receptors.

Agent	Company	Molecule	Current status
Efgartigimod	ARGENX	Humanized IgG1 Fc Fragment	Approved for treatment of AChR+ gMG
Rozanolixizumab	UCB	Humanized IgG4 Monoclonal	Phase 3 completed
Nipocalimab	Johnson and Johnson	Humanized aglycosylated monoclonal ab	Phase 3 study ongoing
Batoclimab	Immunovant	Humanized IgG1 monoclonal ab	Phase 3 study ongoing
ABY-039	Affibody	Bilvalent antibody	Phase 1 study was prematurely terminated

### Efgartigimod

Efgartigimod is a modified Fc fragment derived from human IgG1. It has been specifically engineered to enhance its binding affinity to FcRn receptors under both acidic and physiological pH conditions while maintaining its pH-dependent properties. Flow cytometry and microscopic analysis suggested an augmented affinity and/or avidity of efgartigomod toward the FcRn receptors due to the demonstration of an elevated concentration of efgartigomod specifically within FcRn-positive compartments within cells, concomitant with enhanced lysosomal accumulation.

#### Phase 1 study

Ulrichts et al. conducted a double-blind, placebo-controlled phase 1 study on 62 healthy volunteers (61). There was an enhanced clearing of IgG1 levels following multiple ascending doses (MAD)of intravenous (IV) efgartigomod compared to administration of IV single ascending dose (SAD), with a 50% reduction in the IgG levels following SAD and a 75% reduction in IgG levels following MAD of efgartigimod. Following the last infusions of efgartigimod, IgG levels returned to their baseline levels after approximately 8 weeks. There were no notable alterations in the IgA, IgE, IgM, and IgD levels, indicating no significant impact on these immunoglobulin subclasses. Additionally, efgartigimod did not interfere with albumin homeostasis. The drug had no safety concerns, with most adverse effects being mild and self-limiting and most side effects being seen at higher doses. Mild headache was the most common side effect encountered, which subsided with minimal intervention. In the phase 1 study, no significant production of anti-drug antibodies was observed.

#### Phase 2 study

In the phase 2 exploratory study, Howard et al. conducted a multicenter, randomized, double-blind, placebo-controlled trial of anti-ACR positive generalized myasthenia gravis (ACHR+ gMG) subjects. This study represents the first investigation providing a clinical profile of Fc receptor antagonism in generalized myasthenia gravis (gMG) (62).

The study included participants who had impaired daily activities of living, as indicated by an MG-ADL score of 5 or higher at screening and baseline, with more than 50% of the score attributed to nonocular items and was diagnosed with MGFA class II-IVa disease. Individuals who had a recent malignancy or thymectomy within 3 months of screening were excluded. A total of 24 patients were randomly assigned to receive 10 mg/kg IV efgartigimod or a matched placebo in a 1:1 ratio for a total of 4 doses over 3 weeks, in addition to their standard-of-care therapy. The study aimed to assess the impact of efgartigimod on patient safety and the effectiveness of the treatment in managing ACR+ gMG. The secondary endpoints included an assessment of efficacy based on the improvement in the outcome measures at week 11. In addition, pharmacokinetics (PK), pharmacodynamics (PD), and immunogenicity were evaluated. The scales deployed to assess the efficacy, including the quantitative myasthenia gravis score (QMG), myasthenia gravis activity of daily living (MG-ADL), myasthenia gravis composite disease scores (MGC) and the revised 15-item MG-quality of life (MG-QoL15r) (Table 2).

**Table 2:** Methodology of the phase 2 and phase 3 results.

Therapeutic name	Phase	Treatment arms	Inclusion	Sample size	Route and dose of administration	Primary end-points	Secondary endpoints
Efgartigimod	2	Double blinded RCT (1:1).	AChR+gMG MG-ADL ≥ 5 MGFA II-IV	24	10 mg/kg efgartigomod or placebo IV wkly 4 doses over 3 wks	Safety and efficacy	Change from baseline to 11 weeks: QMG MG-ADL MGC MGQoL15r
Efgartigimod	3	Double blinded RCT (1:1)	gMG MG-ADL ≥ 5 MGFA II-IV	167	10 mg/kg efgartigomod or placebo IV wkly 4 doses and then repeat dosing if needed	MG-ADL responders.	QMG responders Early MG-ADL responders
Rozanolixizumab	2	Double blinded 2 Period RCT (1:1).	gMG QMG ≥11	43.	Period 1: 7 mg/kg rozanolixizumab. or placebo Qwkly for 3wks. Period 2: 7 mg/kg or 4 mg/kg Rozanolixizumab Qwkly for 3wks	Change from baseline to day 29: QMG.	Change from baseline to day 29: MG-ADL MGC Safety
Rozanolixizumab	3	Double blinded RCT (1:1:1)	ACHR+ MG Musk+MG MGFA II-IVa MG-ADL ≥ 3	200	7 mg/kg Qwkly or 10 mg/kg Qwkly of rozanolixizumab for 6 wks.	Change from baseline to day 43: MG-ADL	Change from baseline to day 43: MGC QMG
Nipocalimab	2	Double blinded 5 arm RCT (1:1:1: 1:1)	gMG	68	5 mg/kg q4wkly 30 mg/kg q4wkly 60 mg/kg q2wkly 60 mg/kg q2wkly, or placebo q2wkly for 8 weeks	Change from baseline to day 57 of MG-ADL Effect on total IgG and anti-ACHR ab level	Change from baseline to day 57: QMG, MG-QoL15r
Nipocalimab	3	Double blinded RCT (1:1)	AChR+ gMG MGFA II-Iva MG-ADL ≥ 6		30 mg/kg at first infusion, 15 mg/kg thereafter Q2wks for 24 wks	Change from baseline to wks 22,23,24 of MG-ADL	
Batoclimab	2	Double blinded 3arm RCT (1:1:1) with OLE	AChR+ gMG MGFA II-Iva QMG score ≥ 12	15	Batoclimab 680 mg, Batoclimab 340 mg, Placebo Qwkly SC for 6wks	Safety and efficacy	Change from baseline to day 43: QMG MG-ADL MG-QoL15r
Batoclimab	3	Quadruple blinded 3 arm 2 period RCT (1:1:1) with OLE	gMG MGFA II-Iva QMG score ≥ 11 MG-ADL ≥ 5		Period 1: Batoclimab 680 mg, Batoclimab 340 mg, Placebo Qwkly SC for 6wks. Period 2: Batoclimab 340 mg QWkly, Batoclimab 340 mg Q2Wkly, Placebo Qwkly SC for 6wks	Change from baseline to 12 wks of MG-ADL In ACHR+MG	Change from baseline to 12 wks of QMG in ACHR+MG Change from baseline to 24 wks of MG-ADL, QMG in ACHR+MG

Efgartigimod was reported to be safe and well-tolerated by all the patients who received the drug and had no serious adverse effects (SAE) or severe treatment-emergent adverse effects (TEAE) reported with no significant difference in the side effect profile between the efgartigimod group compared to the placebo group. No incompatibility was seen between efgartigimod and the standard of care therapy used in MG. Reduced monocyte count and headaches were the most frequent side effects noted in the study, with most reported side effects being mild in severity. Other side effects included rhinorrhea, pruritis, injection site reaction and herpes zoster infection. Of note, the patient diagnosed with herpes zoster was already on prednisone and mycophenolate mofetil, and the authors were uncertain if efgartigimod was the causative factor leading to herpes zoster.

Patients who received efgartigimod demonstrated clinically meaningful and sustained improvement, consistently observed across all clinical scales, including MG-ADL, QMG, MGC, and MG-QoL15r (Table 3). Maximum clinical improvement was observed 1 week following the final infusion dose and persisted even after stopping the medication. Maximum mean changes in efgartigimod vs. placebo for QMG score (−5.7 vs. −2.1), MG-ADL (−4.4 *VS* -2.9), MGC (−9.4 vs. −4.4) and MG-QoL15r (−6 vs. −2.1). 75% of the patients treated with efgartigimod obtained a clinically significant, statistically significant improvement in MG-ADL for a period of 6 weeks. In contrast, only 25% of patients in the placebo group showed similar improvement. There was evidence of rapid reduction of total IgG and IgG subtypes in all patients who received efgartigimod. A reduction of up to 40% was seen after the first administration, and a maximum reduction of up to 70% was seen around 1 week after the last infusion. Reduction in anti-ACHR ab levels mimicked the reduction in total IgG levels. The clinical benefit correlated with the initial drop in IgG levels, but the clinical effects persisted for 8 weeks even when the IgG levels returned almost to baseline.

**Table 3:** Summary of the phase 2 and phase 3 results.

Therapeutic drug	Phase	Primary end point results (Mean change from baseline)	Secondary end point results (Mean change from baseline)	Adverse effects
Efgartigimod	2	Safe and well tolerated	QMG −5.7 MG-ADL −4.4 MG-QoL15r −6	No serious side effects Most common side effects: headaches, rhinitis, pruritis
Efgartigimod	3	MG-ADL responders (68%)	QMG responders (63%) Early MG-ADL responders (57%)	5% patients had serious side effects. Most common side effects: headaches, nasopharyngitis
Rozanolixizomab	2	QMG −1.8	MG-ADL −1.8 MGC −3.1	No serious side effects Most common side effects: headaches
Rozanolixizomab	3	7 mg/kg MG-ADL −3.70 10 mg/kg MG-ADL −3.40	7 mg/kg MGC -5.93, QMG −5.40 10 mg/kg QMG −6.67, QMG −7.55	No serious side effects Most common side effects: headaches diarrhea, nausea, pyrexia
Nipocalimab	2	30 mg/kg q4wkly MG-ADL −3.7 60mh/kgQ2wkly		No serious side effects Most common side effects: diarrhea, headache, nasopharyngitis
Batoclimab	2	Safe and well tolerated 680 mg SC Total IgG −776.1. 340 mg SC Total IgG −59.3	MG-ADL −3.8 QMG −3.9 MGC −8.0	No serious side effects

#### Phase 3 study

The ADAPT trial was a phase 3 randomized multicentre, double-blinded, placebo-controlled trial of efgartigimod in patients with gMG with or without anti ACHR antibodies with the disease classified as class II-IVa as per MGFA classification with MG-ADL of at least 5 with more than 50% of the score attributed to nonocular symptoms (63). The study excluded patients with thymectomy within 3 months of screening. Patients were randomized in a 1:1 ratio to receive efgartigimod or placebo. The patient received the same dose of efgartigimod of 10 mg/kg IV or matching placebo as four weekly infusions per cycle. After each cycle, there were at least 5 weeks of follow-up, and retreatment was possible if the patient was a clinical responder with an MG-ADL score of ≥5 and no longer had a meaningful clinical response. Clinically meaningful improvement was defined as a reduction in ≥2 points in MG-ADL compared to the baseline sustained for ≥4 weeks, the first improvement occurring in the 4th week of the 1st cycle. The retreatment was possible no sooner than 8 weeks. In the study period of 26 weeks, a maximum of 3 cycles were possible. The study primarily assessed the proportion of ACHR-positive patients who were MG-ADL responders in the first treatment cycle.

Secondary outcome measures encompassed evaluating the proportion of individuals exhibiting a favourable response in terms of the QMG score (clinically meaningful improvement was defined as ≥3 point reduction, with the first improvement occurring the 4th week of the 1st cycle), percentage of MG-ADL responders in the overall population after cycle 1, the proportion of time patients showed a meaningful response in ACR+ gMG patients up to 126 days, time from last infusion of cycle 1 to no longer have a clinically meaningful response in ACHR positive patients and proportion of early MG-ADL responders (first MG-ADL response of reduction in ≥2 points from baseline occurring within the first 2 weeks of the cycle 1) in ACHR+ gMG patients (Table 2).

167 patients were enrolled in the trial, with 129 ACHR+ gMG. 68% of patients in the efgartigomod group achieved all primary outcomes compared to the 30% in the placebo group. Secondary endpoints were met in the efgartigimod group with improvement in outcome measures which was statistically significant (Table 3). There was a greater percentage of QMG responders (63%) compared to placebo (14%), MG-ADL responders (all patients) in cycle 1 (68%) compared to placebo (37%), early MG-ADL responders (57%) compared to placebo (25%) and there was a clinically meaningful improvement in MG-ADL score for the efgartigimod group 48.7% of the time compared to placebo 26.6%. The drug was safely tolerated, with no deaths associated with the administration of the efgartigomod. Most TEAEs were mild to moderate; however, 5% of patients reported serious adverse effects. The most common adverse effect reported following efgartigimod administration was headaches, followed by nasopharyngitis. The IV formulation of the drug was approved by FDA, EMA and in Japan for use in patients with ACHR+ gMG (Table 3). A different route of administration, such as subcutaneous therapy, may be effective for MG, lessen the side effects and allow treatment to be continued.

#### ADAPT+ study

Of the 167 patients enrolled in the ADAPT trial, 151 (91%) entered the ADAPT + trial, an extended open-label trial assessing the safety and efficacy of efgartigimod. 106 ACHR+ gMG and 33 patients with anti-acetylcholine receptor antibody-negative generalized myasthenia gravis (ACHR- gMG) were included, of which 66 were previously in the placebo group (64). A dose of 10 mg/kg of efgartigimod was given IV, following a treatment schedule consisting of once-weekly infusions for a period of 4 weeks. Subsequent treatment cycles were determined based on clinical evaluation. Throughout the study, patients received an average of 5.1 treatment cycles comprising 20.5 infusions. The median duration of participation in the study was 371 days, resulting in a cumulative observation time of 138 patient-years. Efficacy was assessed during each cycle utilizing MG-ADL and QMG scales. The mean change in the baseline for MG-ADL was −5.1, and for QMG was −4.7, suggesting the efficacy of long-term treatment with efgartigimod consistent across multiple cycles with no safety concerns identified, with most of the AE being mild to moderate.

### Rozanolixizumab

Rozanolixizumab is a human anti- FcRn IgG4 antibody having a high affinity to the FcRn receptors (65). The drug was first studied in animals and was reported to be well tolerated, with no mortality or serious side effects following its IV or subcutaneous (SC) administration. There was a 75–90% reduction in IgG levels, with the maximum effect seen on day 10. No susceptibility to increased infections or raised acute phase reactants was noted in patients who received the drug. There was no change in serum concentration of IgA and IgM levels; however, a small decrease in albumin levels was observed, most likely due to steric hindrance due to bound antibodies. There was a treatment-related effect on the relative or absolute number of lymphocyte counts on immunophenotyping (65, 66).

#### Phase 1 study

This study investigated the dose escalation of IV or SC rozanolixizumab in healthy individuals (66). A total of 49 patients were subjected to randomization, where they were assigned to receive either rozanolixizumab or a placebo. The doses administered were 1 mg/kg, 4 mg/kg, and 7 mg/kg. The drug was administered as a single infusion over 1 h. The 7 mg/kg SC route administration had a better safety profile and tolerability than the IV group. After IV drug administration, there was a dose-dependent increase in side effects of headache, vomiting, nausea, and pyrexia. 4 severe TEAEs were reported following higher doses of the IV formulation. There were no severe side effects reported in patients who received SC drugs. A dose-dependent decrease in IgG levels was similar in both IV and SC groups. There was a 48% reduction in IgG levels at the highest dose of IV formulation and a 43% reduction in IgG levels following SC formulation. The reduction in serum IgG levels was maximum at day 7–10, persisted for weeks and gradually returned to baseline.

#### Phase 2 study

This multicenter, double-masked, placebo-controlled trial was conducted in two periods. The subjects were randomized to receive either three once-weekly SC infusions of rozanolixizumab at a dosage of 7 mg/kg or a placebo in the initial period (67). Following a two-week drug-free interval, patients were re-randomized in the second period to receive rozanolixizumab at either 7 mg/kg or 4 mg/kg, and an observation period between days 44 and 99 followed the second treatment period.

The study primarily evaluated the change in QMG on day 29. Secondary objectives included assessing the change in MG-ADL and MGC at day 29, responder rates (defined as a reduction of at least 3 points) in the MG scores and monitoring the safety profile of rozanolixizumab. Patients at least 18 years of age diagnosed with generalized MG with positive anti-ACHR antibody titre or anti-Musk antibody titre with a QMG score of at least 11 or more with eligibility for IVIG or PLEX indicating moderate to severe disease were included in the study. Patients with a thymectomy within 6 months of screening were excluded from the study. Of the 43 patients randomized, 21 patients received rozanolixizumab, and 22 received a placebo (Table 2).

At day 29, the mean change in QMG score from baseline was −1.8 for rozanolixizumab compared to −1.2 for placebo. For MG-ADL, the mean change was −1.8 for rozanolixizumab and − 0.4 for placebo. For the MGC score, the mean change was −3.1 for rozanolixizumab and − 1.2 for placebo. Although the rozanolixizumab group showed an overall improvement in outcome measures compared to the placebo group from baseline to day 29, the difference did not reach statistical significance. On day 29, the responder rates were higher in patients receiving rozanolixizumab 7 mg compared to placebo for QMG (38% vs. 23%), MG-ADL (48% vs. 14%), and MGC (48% vs. 27%). Interestingly, even greater responder rates were observed on day 22 for QMG scores (52%) and MGC scores (55%) compared to day 29. The reduction in QMG score reached its lowest point on day 21 and returned to baseline on day 29, just before the start of period 2. The short subcutaneous treatment duration could be one of the reasons that the primary endpoint was not achieved on day 29. The continuation of rozanolixizumab treatment at a 7 mg/kg dose demonstrated further improvements in outcome measures during period 2. Notably, the nadir of QMG and MG-ADL scores was observed on day 21 after reinitiating rozanolixizumab at the 7 mg/kg dosage. However, by the end of the 99-day observation period, all the measured outcomes returned to their baseline values. These findings suggest that the mode of action of the drug was reversible (Table 3).

There was a moderate reduction in the assessed outcome measures in subjects who received 4 mg/kg of rozanolixizumab with a sustained reduction in QMG score until day 78. Of note, both groups demonstrated dose-dependent improvements in the measured outcome measures when compared to the placebo group.

In period 1, a rapid decline in IgG levels (52%) was seen compared to placebo (4%) at day 29. Nadir in IgG level in the rozanolixizumab group occurred on day 22 (61%). In period 2 of the study, there was a dose-dependent decrease in IgG levels observed among the groups that received Rozanolixizumab at doses of 7 mg/kg and 4 mg/kg, in comparison to the placebo group.

The most commonly encountered adverse effects were mild to moderate headaches (57% rozanolixizumab 7 mg/kg group vs. 14% in the placebo group), which responded well to standard therapy. There were no serious infections or opportunistic infections noted.

#### Phase 3 study

MycarinG study was a large multicentre trial compared SC administration of 7 mg/kg, 10 mg/kg of rozanolixizumab or placebo for 6 weeks in patients with ACHR/MuSK positive MG (68). The study incorporated subjects with MGFA class II-IVa, with QMG scores exceeding 11 and MG-ADL scores of at least 3. The primary endpoint of the study aimed to evaluate the change in MG-ADL scores from baseline to day 43. The secondary endpoints assessed the changes in MGC, QMG, and Myasthenia gravis patient-related outcomes (MG-PRO) from baseline to day 43 (Table 2). 200 patients were enrolled in the study. There were statistically significant and clinically meaningful improvements in the various outcome measures used in both 7 mg/kg and 10 mg/kg doses (69). For the dose of 7 mg/kg vs. placebo mean change from baseline to day 43 for MG-ADL (−3.37 vs. −0.78), MGC (−5.93 vs. −2.03), and QMG (−5.40 vs. −1.92). For subjects with 10 mg/kg group vs. placebo change in baseline to day 43 for MG-ADL (−3.40 *vs*-0.78), MGC (−7.55 vs. −2.03) and QMG (−6.67 vs. −1.92). 72% of patients in the 7 mg/kg group were MG-ADL responders, and 69% of patients in the 10 mg/kg group were MG-ADL responders. The study was not statistically powered to compare the two doses of rozanolixizumab. Both doses were well tolerated, and no severe side effects were seen, with the most frequent TEAE being headaches followed by diarrhea and pyrexia (Table 3).

## Rozanolixizumab is currently awaiting approval for treatment in MG

### Nipocalimab

Nipocalimab is a glycosylated fully human monoclonal antibody of the IgG1 class that exhibits high affinity for FcRn exhibiting pico affinity to FcRn at both endosomal and extracellular pH levels. This molecule shows selective binding to, saturation of, and blocking of the IgG binding site on the endogenous FcRn. As a result, it inhibits the FcRn-mediated recycling of IgG, reducing pathogenic IgG levels with no impact on IgG production. One of the pharmacokinetic properties of the drug is the minimal transfer of the drug across the human placental lobule so that it does not reach the fetal circulation (70).

#### Phase 1 study

The Phase 1 study was designed as a two-part ascending dose study (71). Part 1 studied SAD up to 60 mg/kg, and Part 2 studied MAD of 15 or 30 mg/kg weekly for 4 weeks. 50 healthy volunteers were recruited for the study. The single dose of nipocalimab resulted in dose-dependent serum IgG levels reduction similar across all IgG classes. The single dose of nipocalimab at 30–60 mg/kg doses maintained serum IgG levels at or below 50% of the baseline for 18 and 27 days, respectively. Multiple doses of 15 to 30 mg/kg achieved a mean reduction in IgG levels by approximately 85% of the baseline and maintained levels below 75% or more for up to 24 days. There was no effect on other immunoglobulins such as IgM, IgA, IgE or other inflammatory cytokines. At a higher dose, there was a mild reduction in albumin noted. The drug was well tolerated in all cohorts, with no deaths, infusion site reactions, systemic allergic reactions or severe TEAEs noted. Due to this pharmacokinetic property, the minimal transfer of the drug across the human placenta may help treat patients in the reproductive age group where teratogenicity and fetal health are of major concern. There is a further exploration of this agent in pregnant women at risk of autoimmune hemolytic disease of newborns (72).

#### Phase 2 study

Vivacity-MG is a phase 2 multicenter study that employed a randomized, double-blinded placebo trial design to assess the efficacy of nipocalimab in moderate-to-severe gMG (73). A total of 68 patients were enrolled and randomly assigned in a 1:1:1:1:1 ratio to receive various intravenous doses of nipocalimab (5 mg/kg every 4 weeks, 30 mg/kg every 4 weeks, a single dose of 60 mg/kg, 60 mg/kg every 2 weeks) or placebo every 2 weeks during the 8-week treatment period. The primary outcome measure of the study was the change in the MG-ADL score from baseline to day 57. The secondary endpoints included assessing the changes in the QMG score and MG-QoL15r at day 57 (Table 2).

The maximum and most consistent reductions were observed in subjects in the nipocalimab 30 mg/kg q4w and 60 mg/kg q2w treatment groups compared to placebo for MG-ADL (−3.7 vs. −1.3) even though statistical significance was not reached. Following single-dose administration of 60 mg/kg, the mean change from baseline to day 29 vs. 57 for MG-ADL was (−3.9 vs. −1.5), indicating the effect of a single dose of nipocalimab may not last for more than 1 month. In contrast to the placebo group, it was observed that 52% of patients who received nipocalimab across all four dosing regimens experienced a substantial and long-lasting reduction in MG-ADL (a change of ≥2 points for at least four consecutive weeks). Only 15% of participants in the placebo group exhibited a similar response. Clinically meaningful changes were observed in a rapid timeframe, with noticeable improvements occurring within 2 weeks. A dose-dependent significant reduction of the total serum IgG and anti-ACR antibody titres significantly correlated with the MG-ADL improvement. The maximum reduction in serum total IgG levels of 80% was seen following the highest dose of 60 mg/kg q2wkly administration. There was a reduction in total IgG 1 week following the first infusion, which returned to baseline 8 weeks after the last infusion.

The drug had a favourable safety outcome with no significant adverse effects. There was an equal incidence of infections and headaches in the patients who received nipocalimab and placebo. No adverse side effects led to treatment discontinuation in any of the groups. Similar results for albumin were observed in healthy participants with dose-related, self-limiting reductions and was maximum with a dose of 60 mg/kg q2wkly. The most common TEAE reported were diarrhea, headaches and nasopharyngitis (Table 3).

#### Phase 3 study

An ongoing phase 3 multicenter study is currently being conducted to assess the efficacy, the safety profile of nipocalimab in adult patients with seropositive gMG (74). Inclusion criteria include patients diagnosed with gMG classified as MGFA class II-IVa and have an MG-ADL of 6 or higher. Individuals who underwent thymectomy within 12 months of screening are excluded from the study. Participants in the study were randomly assigned in a 1:1 ratio to receive either nipocalimab or a placebo. The administration of nipocalimab consisted of an initial infusion of 30 mg/kg, followed by subsequent infusions of 15 mg/kg, given once every 2 weeks for a total duration of 24 weeks. The placebo group received corresponding intravenous infusions at the same intervals. The study will primarily ascertain the efficacy of Nipocalimab compared to placebo, using MG-ADL over weeks 22, 23, and 24 (Table 2).

### Batoclimab

Batoclimab is a fully human IgG1 monoclonal antibody which can be used for SC or IV administration.

#### Phase 1 study

A phase 1 study (RVT-1401-1,001) investigated the effects of batoclimab on serum total IgG and albumin levels (75). This trial included IV and subcutaneous SC administration of batoclimab at various doses and durations. Batoclimab effectively reduced total IgG concentrations in a dose-dependent and reversible manner. The maximum reductions ranged from 13 to 67% for single IV doses and 14 to 48% for single SC doses. The SC weekly dose groups showed low inter-subject variability in total IgG reduction. The 680 mg dose exhibited a more consistent effect than the 340 mg dose. The weekly SC dose of 680 mg led to a total IgG reduction of 78%, consistent with other anti-FcRn agents in development. The time course of total IgG reduction was independent of the route of administration (IV or SC) and dose. However, the time to return to baseline was dose-dependent, ranging from 29 to ≥85 days for single doses and 21 to 24 days for weekly SC doses. Serum total IgG concentrations returned to normal levels for most subjects after discontinuing dosing. Batoclimab had a lesser impact on albumin levels compared to total IgG. Albumin reductions were 22 and 34% for the weekly SC dose groups. However, albumin levels returned to normal within a few weeks after the last dose.

A similar phase I randomized controlled trial was conducted in the Chinese population, where healthy subjects were given a single subcutaneous dose of batoclimab or placebo in doses of 340 mg, 510 mg, 680 mg, with an equal allocation ratio of 1:1:1:1 (76). The participants were followed up for 85 days. Twenty-four subjects were included in the study. There was evidence of a rapid dose-dependent reduction in total IgG levels, reaching a nadir at day 11 with the steady recovery of the IgG levels from day 11 to day 85. No serious side effects were reported, and the most reported illness in subjects who received the drug was an influenza-like rash.

#### Phase 2 study

A phase IIa clinical trial employing randomization, double-blinding, and placebo control, investigated the safety and effectiveness of subcutaneous administration of batoclimab (77). Subjects with ACHR + gMG with (MGFA) Class II-IVa and QMG score ≥ 12 baselines were included in the study. Subjects with a thymectomy in the past 12 months were excluded. Patients included in the study were assigned to one of three groups receiving a weekly dose of batoclimab (680 mg), a weekly dose of batoclimab (340 mg), or a placebo for a duration of 6 weeks. Following this initial phase, an open-label extension was initiated, during which patients received batoclimab (340 mg) on days 50, 64, and 78. Assessment of safety and efficacy was the primary endpoint of the study, measured by percentage changes in total IgG levels. Secondary endpoints included QMG, MG-ADL, and MGC scores, with a change in scores on day 43 from baseline (Table 2).

Improvement in all the outcome measures, including MG-ADL scores, was reported on day 43. There was a dose-dependent rapid and sustained decline in the IgG levels with a − 76.1% change in IgG levels with the dose of 680 mg QWkly treatment arm, −59.3% change in 340 mg SC treatment group compared to a 1.5% change in the placebo group. The mean change in QMG at day 43 from baseline for batoclimab vs. placebo was (−3.8 vs. +0.6), MG-ADL (−1.8 *VS* -0.4) and MGC (−8.0 vs. +1.4). In the group receiving batoclimab, the MG-ADL responder rates, which indicate the percentage of patients with a > 2-point improvement, were 60%. In contrast, the placebo group had a responder rate of 20%. The maximum benefit from the treatment was reported 4 weeks after the initial intervention, and this benefit was sustained for a period of 3 weeks. Both doses of the drug were found to be safe and well-tolerated. The reduction in the pathogenic Ig levels correlated with the clinical benefit (Table 3). Adverse effects were mild to moderate, with no serious adverse effects or death (3).

In another phase 2 trial conducted in China, the tolerability and effectiveness of subcutaneously administered batoclimab were evaluated in patients with moderate to severe gMG. Eligible patients were randomly assigned to receive either batoclimab (680 mg), batoclimab (340 mg), or a placebo weekly for 6 weeks. Following this, there was an open-label extension phase where all patients received batoclimab (340 mg) weekly for another 6 weeks. The primary aim of the study was to determine the change in MG-ADL score, measured from baseline to day 43, as the main objective. On day 43, significant improvements were observed in all outcome measures, including MG-ADL scores. Top-line results indicated that both administered doses of batoclimab exhibited prompt and substantial reductions in disease severity, as evaluated by the MG-ADL, in a manner that was clinically and statistically significant when compared to the placebo. Furthermore, the drug demonstrated a favourable safety profile, with most reported adverse events being of mild intensity and generally well tolerated by the patients (78).

#### Phase 3 study

Study IMVT-1401-3,101 is an ongoing Phase 3 pivotal multicenter, randomized, quadruple-blind, two-period placebo-controlled study done to assess the effectiveness and how safe batoclimab is as induction and maintenance therapy in adult participants with gMG (79). Patients with mild to severe disease with an MG-ADL of ≥5 at baseline were enrolled in the trial. Other inclusion criteria included MGFA Class II-IVa and QMG score of ≥11 with a ≥ 50% score attributed to nonocular symptoms will be included in the study. Patients with a thymectomy in the past 6 months will be excluded from the study. In Period 1, subjects will be randomized 1:1:1 to receive batoclimab 680 mg SC once a week or 340 mg SC once weekly or a placebo as induction therapy. Primary endpoints of ≥2-point improvement in MG-ADL from baseline to week 12 will be assessed. Re-randomized (1:1:1) subjects will receive batoclimab 340 mg SC once weekly or 340 mg SC Q2weeks or receive placebo treatment according to their treatment assignment in Period 1 and the change from Period 1 baseline in MG-ADL score (< 2-point improvement or ≥ 2-point improvement from Period 1 baseline) at Weeks 10 and 12. Only those participants who demonstrate a ≥ 2-point improvement in MG-ADL score from Period 1 baseline during at least one of the 2 final visits of either Period 1 or Period 2 will proceed to enter the long-term extension, which will extend for 52 weeks. The primary endpoint of the study involved evaluating the change in MG-ADL score from baseline to week 12. The secondary endpoints included assessing the change in QMG scores from baseline to week 12 and MG-ADL scores from baseline to week 24. The overall duration of the study is expected to be approximately 80 weeks (Table 2).

#### Phase 1 study

ABY-039, a bivalent antibody-mimetic, has completed a phase 1 trial involving healthy volunteers. It specifically targets the neonatal Fc receptor (80). It has a potent effect on lowering plasma IgG titers in healthy volunteers in Phase 1. ABY-039 program was terminated in June 2020 due to tolerability observations that would limit the target product profile of high subcutaneous doses once monthly maintenance injections.

## Discussion and conclusion

The FcRn blocking agents have been recently developed and have a targeted approach for MG treatment. Given the rapid reduction in pathogenic IgG autoantibodies, many authorities consider this treatment to be medical plasma exchange. The drugs included in this class have been found to be safe and efficacious in the various phase 1, phase 2 and phase 3 trials conducted thus far. FcRn receptor inhibitors demonstrate efficacy across diverse subgroups and a wide range of MG severity, supported by robust clinical evidence. Efgartigimod has been approved in the management of gMG, rozanolixizumab is under review by health authorities, and there are phase 3 trials ongoing for nipocalimab and batoclimab. The optimal dosage and duration of treatment in managing MG still need to be better understood, and more research is needed. Long-term safety, cost of the medications, and optimal usage are some of the factors influencing the use of these newer agents and requiring their judicious use in an appropriate clinical scenario. Overall, the FcRn blocking agents have an exciting and promising role in the management of MG.

## Author contributions

ViB: writing, review, and editing. VeB: writing, review, editing and supervision. All authors contributed to the article and approved the submitted version.

## Conflict of interest

VeB has been a consultant for: Grifols, CSL, UCB, Argenx, Takeda, Alnylam Octapharma, Pfizer, Powell Mansfield Inc., Akcea, Ionis Immunovant, Sanofi, Momenta (J&J), Roche, Janssen, AZ-Alexion, NovoNordisk, Japan tobacco. VeB had research support from: AZ-Alexion, Grifols, CSL, UCB, Argenx, Takeda, Octapharma, Akcea, Momenta (J&J), Immunovant, Ionis.

The remaining author declares that the research was conducted in the absence of any commercial or financial relationships that could be construed as a potential conflict of interest.

## Publisher’s note

All claims expressed in this article are solely those of the authors and do not necessarily represent those of their affiliated organizations, or those of the publisher, the editors and the reviewers. Any product that may be evaluated in this article, or claim that may be made by its manufacturer, is not guaranteed or endorsed by the publisher.
